# Integrating Bioprinting and Increased Throughput: Next-Generation Models for Cardiac Research

**DOI:** 10.3390/ijms262311589

**Published:** 2025-11-29

**Authors:** Stephanie Nguyen, Zachary Laksman

**Affiliations:** 1 School of Biomedical Engineering, University of British Columbia, Vancouver, BC V6T 2B9, Canada; 2 Department of Medicine, University of British Columbia, Vancouver, BC V6T 1Z3, Canada; 3 Centre for Heart Lung Innovation, Vancouver, BC V6Z 1Y6, Canada

**Keywords:** integration, high-throughput, 3D bioprinting, 3D cell culture, cardiac tissue, models

## Abstract

Over the past two decades, three-dimensional cell culture (3DCC) and bioprinting (3DBP) technology have been at the forefront of developing engineered human cardiac tissue. Compared to 2D culture systems, 3DCC-based models more accurately replicate key characteristics of living tissues such as diffusion dynamics, interactions between cells and the extracellular matrix, as well as the presence of supporting stromal components. The rise of new 3DBP techniques serves to facilitate the robust and rapid generation of 3D tissue alongside real-time assessment of their characteristics. In order to capitalize on their translational potential, high-throughput screening (HTS) is required as research draws nearer to replicating clinical trials with cardiovascular-bioprinted tissues, and testing for the safety and efficacy of novel therapeutics. In this review, we summarize trending cardiac tissue models, as well as the state of their related or integrated HTS process and development. With an emphasis on the need for scale-up, compatibility, and standardization in HTS, the coalescence of 3DBP models and screening can provide improved disease modeling, drug efficacy, and toxicity testing.

## 1. Introduction

Cardiovascular diseases (CVD) remain the global leading cause of mortality as of 2024 [1], with projections of a 90.0% increase in prevalence and a 73.4% increase in mortality between the years 2025 and 2050 [2]. These diseases manifest through pathological changes that result from structural changes and damaged biochemical pathways [3], which lead to the progressive impairment of overall cardiovascular function. The lack of endogenous cardiac stem cells in adult individuals limits the heart’s intrinsic regenerative capacity when there is prolonged ore recurrent heart damage [4]. As a result of chronic heart damage, individuals may experience acute, irreversible reduction in heart function, or ejection fraction, which manifest clinically in the form of arrhythmias and heart failure [5].

Current treatments are mainly palliative, with heart transplantation or mechanical circulatory assist devices remaining the most viable option in cases of end-stage heart failure [6,7]. Both treatment options are limited, both in terms of indication and availability. Heart transplantation programs in particular have suffered from donor shortages, immune rejection, and other post-operational complications [6]. Hence, the demand for new sources of cardiac regenerative therapies infused urgency, investment and innovation in the field of cardiac regenerative bioengineering.

One of the earliest and still most employed models are called engineered heart tissues (EHTs). Introduced by Zimmerman and colleagues in 2006 [8,9], EHT generation is an approach that utilizes the cells and matrix proteins found in the heart to recreate the complex tissue and microenvironment in vitro. The novelty stems from the three-dimensional nature of EHTs, which is a closer representation of cardiac tissue, and can employ a number of different cell types, and enables a wider range of assessments, such as proteomic and genomic analyses of disease-specific phenotypes, contractile dynamics, electrophysiology, and tissue microtomy [10]. As a result, studies have shifted to include 3DCC in place of conventional monolayer cell culture systems, with new models utilizing organoids, spheroids, assembloids, and modernized EHTs. Within the realm of 3DCC, 3D human cardiac tissues have been generated for diverse studies, including disease modeling, drug screening, human development, and regenerative medicine [6,7,8,9].

An ongoing challenge in this field relates to the state of maturation of stem cell derived cardiomyocytes, which has a significant impact on their physiology and response to toxins and therapies. Researchers have sought to advance the state of maturity of these cells by utilizing electrical and mechanical stimulation, as past evidence has been shown that 3DCC and EHTs also contribute to develop a more adult-like phenotype [11,12,13,14,15]. Many attempts in generating sufficiently matured EHT models rely on self-assembly, as observed through cell migration in forming architecture resembling the in vivo counterpart [16]. While novel, self-assembly in regenerative medicine and disease modeling can be limited. In the absence of external patterning or structural cues, the morphology of an EHT may have poor reproducibility, may not accurately reflect the in vivo structures, and may also be incompatible with the patient’s specific anatomical structure when attempting to graft it onto the damaged area of interest [16,17,18]. Three-dimensional bioprinting (3DBP) provides a regulated, automated solution to the manual production of EHTs, addressing significant issues related to repeatability, structural integrity, and patient customization. In contrast to manual techniques that depend significantly on operator expertise and may lead to inconsistent tissue shape and cell distribution, 3DBP allows accurate spatial placement of cells and biomaterials, guaranteeing uniform architecture and functionality throughout constructions. By facilitating the incorporation of patient-specific imaging data (e.g., Magnetic Resonance Imaging or Computed Tomography Scan), bioprinted tissues may be customized to align with individual anatomical features, enhancing transplant compatibility and integration with native myocardium. The digital aspect of the procedure facilitates mass repeatability and scalability, which is critical to enabling high-throughput manufacture of standardized tissues for research, drug screening, and prospective clinical applications.

## 2. Technologies Driving High-Throughput Engineered Cardiac Tissue Generation

### 2.1. Types of Bioprinting

In general, for 3DBP, there are 3 main types: jetting-based (i.e., inkjet, laser-assisted), extrusion-based, and vat photopolymerization-based (stereolithography and digital light processing-based). Jetting-based bioprinting comprises various unique categories determined by the mechanism employed to expel bioink droplets. Inkjet bioprinting uses an ejector to dispense continuous droplets of bioink, with the two ways employing either localized heat to eject the ink with a vapor bubble or piezoelectric technology to produce droplets with an electric current [19,20]. One must consider the working frequency of the acoustic waves produced in the use of piezoelectric crystals as its working range of 15 to 25 kHz may harm cells [19,21]. Another concern that researchers may have is that high temperature within the nozzle can reach above 200 °C, which can result in cell impairment and hydrogel denaturation if exposed for an extended duration [22]. However, the exposure time is extremely short (2 µs) and the consequent temperature drop to room temperature occurs in seconds. Thus, the cell viability (>90%) was able to be maintained prior to and following the printing [23,24]. Consequently, thermal inkjet printing technique exhibits greater biocompatibility with biological systems in comparison to piezoelectric printing. The principle technique relies on consistent mechanical force and gravity to produce a 3D structure, with the path of bioink deposition preprogrammed and the base elevator electrically controlled along the *z*-axis [25]. Replicating the spatial architecture of the native heart requires precise heterotypic cell configurations, which inkjet bioprinting provides thanks to its high droplet resolution. Limitations of inkjet bioprinting include the inability to extrude a continuous flow of bioink which may be detrimental to structural soundness [19,26], the requirement to exhibit low cell densities in the bioink to reduce shear stress involved in crosslinking, non-uniform droplet size, low droplet directionality, and nozzle blockages [19].

In laser-assisted bioprinting (LAB), a pulsed laser induces heating in a laser-absorbing layer on a donor slide, resulting in the formation of a microbubble that ejects a precise droplet of bioink onto a receiving substrate on the collector slide [20]. This technique enables high-resolution patterning [27] accommodates viscous bioinks [19], and safeguards cells from shear stress due to its nozzle-free design, rendering it advantageous for tissue engineering and organ fabrication. The printing resolution is influenced by factors including biomaterial viscosity, layer thickness, and laser influence [28,29]. In that sense, control over laser frequency, intensity, motion control, cell density per droplet can be programmed [19,20]. LAB’s exceptionally high spatial resolution (Table 1) makes it useful for capturing the myocardium’s minute structural variations. Some drawbacks of LAB systems are the cost, commercial accessibility, capability of scale-up to large structures, compatibility for deposition of multiple cell types [19,30]. The ability of LAB to deposit several cell types in precisely regulated patterns allows for the restoration of myocardial microdomains, such as CM–fibroblast–endothelial networks. This supports research on paracrine signaling and regulated electromechanical coupling [31].

Extrusion-based bioprinting (EBB) is widely used due to convenience, low cost, its ability for simultaneous usage of multiple biomaterials that can be tunable to match ECM regeneration rate [45], and encapsulate cells and deposit them at physiologically relevant densities [46]. The cell deposition density typically falls between 108 and 109 cells per millilitre [47], which makes it essential for cardiac tissue engineering as cardiac tissues need large numbers of cardiomyocytes per volume. The method relies on constant pressure generated by either pneumatic or mechanical (piston or screw-driven) to extrude a continuous strand of bioinks onto a substrate, thereby forming a 3D construct in a layer-by-layer manner [19,30]. The pneumatic-based technique employs compressed air at a predetermined volume flow rate to drive fluid dispensing systems’ continual extrusion of bio-ink, whereas the piston/screw-based technology mechanically pushes biomaterials out of the nozzle. EBB is categorized into two types: direct and indirect [19]. The bioink in direct EBB polymerizes into a three-dimensional structure that facilitates cell attachment and proliferation. In contrast, indirect EBB employs sacrificial bioink that guides tissue growth and can be removed through thermal or chemical methods. The technique is a viable option for many laboratories since it is extensively used in cardiac tissue studies [48,49], reasonably accessible, and reasonably priced [31]. The disadvantages include low printing resolutions as well as problems in achieving precise cell patterning and organization. Additionally, cell viability may be affected during the gelation or solidification processes following printing [19,50]. Other considerations necessary include the needle diameter as it has a considerable influence on the printed strand diameter and resolution; smaller needles may result in higher resolution strands. In addition, the flow velocity of the biomaterial, the design and accompanying structure formed, process-induced stresses, and the crosslinking of biomaterial solutions are all important factors in bioprinting construction.

The last category of bioprinting—vat photopolymerization-based—relies in the polymerization of photo-cross linkable bioink via ultraviolet (UV) light, infrared light, or visible light beams to solidify light-sensitive, cell-laden bioink and create 3D constructs. This category includes stereolithography (SLA) and Digital Light Processing (DLP). SLA technology has become a leading technique for building complex tissue due to its exceptional resolution and retention of high cell viability [51]. The illumination source to photo-cross link the bioink is a critical component that determines the performance of the SLA process [51]. The characteristics of the illumination source frequently dictate the resolution and accuracy of the printed parts. In addition, the mechanical properties of the scaffold can be adjusted by regulating the illumination conditions [52]. A 3D model generated using Computer-Aided Design (CAD) is converted into a stack of 2D images that represent the cross-sections, which are used as input for the bioprinting system [51]. Each input image is used as a mask and projected onto a thin layer of liquid that is formed on a platform by submerging it in a tank of resin. A laser is directed through a window at the base of the tank, creating cross sections of the three-dimensional construct while selectively polymerizing the biomaterial. Upon completion of one layer, it is extracted from the bottom of the tank, facilitating the flow of fresh resin beneath it. The platform is subsequently lowered, and the procedure is reiterated until the 3D structure is completely printed [19,20].

In contrast to SLA, DLP utilizes a digital light projector as its light source, or a digital micromirror device which comprises hundreds to millions of independently controlled micromirrors [43]. Because the printer head’s x-y directional motion is eliminated, a whole layer may be produced at once, significantly cutting down on printing time, along with material waste [43]. Due to the high resolution and precision of SLA, bioprinting can be scaled to fine details in producing microarchitecturally accurate cardiac microtissues. The main drawback for using both SLA and DLP in printing tissues is the cytotoxic effects from photo initiations and UV light exposure, as well as the high cost of the UV light sources [53,54,55]. The materials used in the DLP method include photopolymer resins, smart photopolymers, hydrogels, ceramic modified resins, and nanocomposite resins [43].

### 2.2. Three-Dimensional Bioprinting of the Heart

Recently, 3DBP technology has shown vast potential and pivotal advantages in producing micro-scale cardiac tissues to overcome the challenges of conventional models. By depositing bioink in a spatial-temporal manner, a high level of control can be achieved in fabricating intricate and complex structures based on digital design. Depending on the bioink formulation, the printed structure can recapitulate different parts of the human heart microenvironment. The precise nature of this technique allows for programmed anisotropy in emulating the structure and alignment of native heart tissue, and in turn, the replicated response and functionality [56,57]. 3D bioprinting facilitates the scalable and reproducible production of tissue constructs, enhancing experimental throughput in various cardiac model systems. In particular, 3DBP can be automated for mass reproducible EHTs in the context of screening multiple drugs. Ultimately, 3DBP showcases the prospect of duplicating patient-specific tissue structures in shape, composition, and function.

As the heart consists of multiple cell types, with the most prominent ones being CMs, cardiac fibroblasts (CFs), smooth muscle cells, endothelial cells (ECs), and peri-vascular cells [58,59,60], bioprinted cardiac tissue constructs would need to comprise a unique blend of cell types to improve biomimicry and for long-term culture [61]. Previously generated tissue constructs with a simple composition of just one cell type have been reported to lack functions that depend on other cell types, structural organization, and integral strength [62]. In another example, a bioprinted cardiac patch utilizing two different types of human cells (Human Umbilical Vein Endothelial Cells, and Mesenchymal Stem Cells) [63] was constructed, then further investigated for wound healing and functional preservation. The combination of the cell types improved cell viability and increased vessel formation at the border zone of the infarcted site in rats with myocardial infarction. This study demonstrated that certain cellular interactions cannot be replicated in a construct unless they are intentionally incorporated into its design. Additionally, the synergistic effect of a heterogeneous mixture consisting of the prominent cardiac cell types in tandem with selected biomaterials can be optimized in order for the print to have similar geometry, topography, biochemical pathways, and mechanical properties of its in vivo counterpart [64].

Several factors play a role in influencing the development, maturation, and overall performance of printed cardiac tissue, such as the physical and biological characteristics of the bioink, intercellular interactions in the extracellular matrix (ECM), and the origin of the cells used. As such, the primary constraint of contemporary 3D bioprinting methods is their reliance on the rheological and bioactive characteristics of the bioinks [65] (p. 8). Hence, considerable effort has been placed into optimizing various formulations of bioink, selecting appropriate cardiac cell types, and bioprinting techniques to design and fabricate different parts of the heart [66,67,68,69]. Various naturally derived and synthetic hydrogel materials are frequently utilized as bioinks, including alginate [70], gelatin [71], collagen I [72], Matrigel [73], fibrinogen [74], hyaluronic acid [75], agarose [76], chitosan [77], and poly (ethylene) glycol (PEG) [78]. These polymers exhibit unique polymerization modes that yield stable scaffolds through an additive process. To date, no hydrogel has been developed or commercialized specifically for standardized bioprinting processes [79]. Several other critical factors must be considered when engineering cardiac constructs, including structural properties like oriented myofibers, comparable mechanical properties, electrical conductivity, and relevant functionalities such as synchronous beating and electromechanical coupling. To evaluate the appropriateness of primary bioprinting techniques for cardiac tissue engineering, it is beneficial to compare their performance (Table 1) regarding resolution, cell viability, structural complexity, scalability, and alignment with essential cardiac-specific requirements (e.g., anisotropic cardiomyocyte alignment, vascularization, contractility).

There is a current focus on generating the myocardium due to its prominent makeup of the heart anatomy, and its vascular network to allow maturation and perfusion of nutrients. In addition, small-scale human hearts, atrial, and ventricular chamber models have also been engineered but not printed [80], with the results showing the maintenance of stable foundation and longevity, and with some models demonstrating physical behaviours, such as contractions [81]. Lee et al. utilized a co-culture of human embryonic stem cell-derived CMs and CFs to bioprint a model of a left ventricle that demonstrated contractile activity by day four of culture, followed by the onset of synchronous beating [82].

The microcirculatory network and macrovessels of the heart are also crucial structures for normal heart function [47,83]. Soft tissue repair involves vascularization, which allows the influx of cells, growth factors, signal molecules, nutrients, oxygen, and drugs to the site of injury [84,85,86]. Thus, ensuring sufficient blood flow to implanted 3D-bioprinted myocardial grafts is essential for maintaining long-term function and cell survival [87]. In creating clinically relevant constructs, the tissue must reach patient-comparable sizes. However, at larger scales, passive diffusion is typically inadequate to supply cells with the oxygen and nutrients they need. In vivo, effective nutrient and oxygen exchange requires that living cells be located within approximately 100–200 μm from a functional capillary [88]. Additionally, the fidelity of disease models for downstream testing is limited without substantial vasculature that facilitates cross-talk across multiple cell types [89]. Indeed, the vascularization of cardiac tissue constructs holds great significance in promoting growth and repair, especially since angiogenesis contributes to cell maturation, organization, and smooth integration of implanted scaffolds [84,86]. This is an opportune problem for 3D bioprinting to solve.

### 2.3. Three-Dimensional Bioprinting Strategies for Next-Generation Tissues

**Figure 1:**
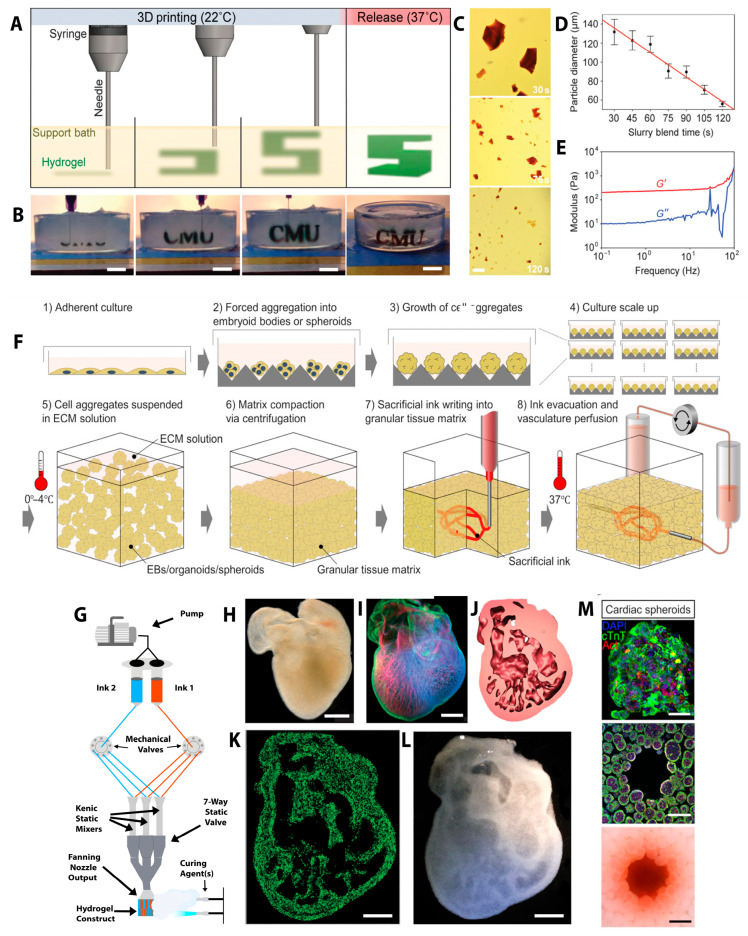
(**A**) A diagram of the FRESH process illustrating the extrusion and cross-linking of the hydrogel (green) within the gelatin slurry support bath (yellow). The 3D object is constructed incrementally, and when completion, is liberated by heating to 37 °C to liquefy the gelatin. Adapted from [97]. (**B**) Images of the letters “CMU” FRESH written in alginate using Times New Roman font (black) and liberated by melting the gelatinous substrate (grey substance in the Petri dish). Adapted from [97]. (**C**) Representative pictures of the gelatin particles created after mixing for 30, 75, or 120 s. Adapted from [97]. (**D**) The red line is a linear fit, while the error bars show standard deviation. The mean Feret diameter of gelatin particles as a function of blending time, from 30 to 120 s. Adapted from [97] (**E**) Rheological examination of the gelatin support bath’s storage (G′) and loss (G″) modulus demonstrates Bingham plastic behavior. Adapted from [97]. (**F**) A detailed stepwise schematic of the SWIFT printing process. Adapted from [98]. (**G**) Ink input selection is automated via the Continuously Extruded Variable Internal Channeling (CEVIC) electric rotary valves feeding into a mechanically controlled Kenics static mixer. The mixer creates alternating channels of the two inks, which leave the nozzle as a structured sheet that curing agents solidify. Adapted from [90]. (**H**) An explanted embryonic chick heart in a dark field microscopy image. (**I**) A confocal microscope view of the 5-day-old embryonic chick heart stained with fibronectin (green), nuclei (blue), and F-actin (red). (**J**) A cross section of the 3D CAD model of the embryonic heart displaying complex internal trabeculation created using the confocal imaging data. (**K**) A cross section of the 3D printed heart in fluorescent alginate (green) depicts the internal trabecular structure from the CAD model. (**L**) A dark-field image of the visible internal structure of a 3D-printed heart. (H-L adapted from [97]). (**M**) Images depicting OBB-based cardiac tissue matrices. Adapted from [99].

Bioprinting techniques can be employed in several manners, contingent upon the specific tissue region and the attributes intended to be replicated in the construct. Multilateral extrusion using microfluidic technology represents an innovative approach to fabricating layered myocardium for anisotropy, hence providing gradients for cellular activity (Figure 1) [90,91,92]. In microfluidic-assisted 3D bioprinting, the bioink is guided through microscale channels that allow precise modulation of flow dynamics [93], component switching [94], and mixing of multiple materials (Figure 1G) [90]. As a result, the morphology, size, and alignment of the printed structures can be finetuned. Further, bioprinting hydrogel microfluidic chips represents a significant and rapidly advancing trend in biomedical research and tissue engineering. This emergent technique facilitates the development of physiologically relevant cardiovascular models by accurately replicating perfusion, shear stress, and three-dimensional cell architecture. This approach supports vascularization, provides PDMS-free biofunctionality, allows for patient-specific geometries, and enhances throughput and reproducibility in drug and disease modelling. Although the target tissue was not cardiac, Bhusal et al. exemplified the ability of the suggested manufacturing procedure to produce microfluidic chips out of biomaterial with various designs and tubing connections [93]. The structure exhibited good fidelity at higher PEGDA concentrations, which made it perfect for connector no-leak attaches. By combining microfluidic control with conventional extrusion-based bioprinting, the printing resolution exhibited significant improvement, surpassing the typical micro-extrusion limit of roughly 50 μm [35]. This hybrid method enables a high level of control of fluid dynamics in both spatial and temporal dimensions. Microfluidic chips primarily consist of channels and fluid reservoirs, which decrease manufacturing costs, waste, chemical reagent usage, and analysis duration [91].

Coaxial nozzles enable the printing of a cell-laden shell, augmented by a sacrificial core to produce hollow filaments that may subsequently be perfused [92]. Shear stress considerations during bioink extrusion are crucial yet adds a layer of complexity as smaller nozzles facilitate higher resolution prints, yet they detrimentally affect cell survival [95]. Coaxial processing offers several advantages, including precise control of concentric multi-material deposition, the capability to use a broader range of printable materials, single-step deposition of sacrificial materials, customizable release profiles, and improved resolution through inline crosslinking [92]. A primary use of coaxial bioprinting is the fabrication of vasculature, which is essential in several tissue engineering approaches. This approach helps minimize shear stress during deposition, largely due to the protective sheath flow that envelops the central laminar stream. Furthermore, coaxial printing enhances print accuracy and mechanical integrity, enabling the creation of softer, contractile cardiac areas in conjunction with stiffer supporting zones—reflecting the anisotropic mechanics of the heart. It also improves throughput and reproducibility by automating the creation of uniform tubular or layered structures, rendering it ideal for next-generation engineered heart tissues, vascularized cardiac patches, and patient-specific transplants.

Embedded bioprinting was developed to address one of the major obstacles of bioprinting, which is the loss of print fidelity and distortion due to gravity [96] as it provides a build chamber filled with a 3D support matrix that envelopes the printed construct as the bioink is deposited. Ergo the incorporation of support baths revolutionized bioprinting by facilitating the utilization of soft biological materials that were previously too fragile to print by providing isotropic stabilization throughout the printing process to decouple the gelation time and cross-linking mechanisms from print fidelity [96]. Freeform Reversible Embedding of Suspended Hydrogels (FRESH) 3D bioprinting is an implementation of embedded bioprinting, developed in 2015 by researchers at Carnegie Mellon University [97] and remains a prominent technique due to its ability to print delicate structures utilizing low-viscosity bioinks. Utilizing a support bath that can reversibly function as both a solid and a liquid, FRESH mitigated the influence of gravity, facilitating the production of intricate, high-resolution tissue scaffolds [96]. This method addresses the limitations of traditional bioprinting by maintaining the geometry of the bioprints as bioink is extruded into a yield-stress support bath, which preserves the position of the bioinks until curing occurs (Figure 1A,B)) [96]. Lower viscosity hydrogels facilitate improved cell spreading, alignment, protection of cells from high shear stresses, and maturation of cardiomyocytes, thereby enhancing the functionality of prints beyond mere structural integrity [46]. Numerous variables, such as the hydrogel being printed and its cross-linking kinetics, the size of the gelatin microparticles (Figure 1C,D), the nozzle diameter, the extruder translation speed, and the flow rate, material deformation deformation (Figure 1E), affect the diameter of the extruded hydrogel filament [97]. Ideally, the slurry used for the bath should exhibit Bingham plastic characteristics which allow for the precise deposition of soft, complex 3D structures that can maintain their shape and support living cells (Figure 1F). Hinton et al. utilized FRESH bioprinting to replicate complex 3D biological structures by imaging, segmenting, and modeling a day-5 embryonic chick heart with intricate internal trabeculations (Figure 1H,I) [97]. The scaled-up heart model was printed in fluorescent alginate, and multiphoton imaging confirmed that the internal architecture closely matched the digital design (Figure 1K,L). Additional comparisons of the model, G-code, and printed construct, along with dark-field imaging, validated the technique’s high fidelity down to submillimeter features.

Efforts to generate enhanced engineered cardiac tissue have concentrated on the development of bioinks aimed at improving tissue electromechanical coupling. This is achieved by incorporating composite materials (e.g., graphene [100], mXene [101], gold nanorods [102], and PEDOT:PSS (poly(3,4-ethylenedioxythiophene) polystyrene sulfonate) [103], that increase conduction velocity and promote synchronous contraction of cardiomyocytes. Hydrogel combinations such as blended decellularized extracellular matrix (dECM) with reduced graphene oxide (rGO) [100] have been evaluated and demonstrated significantly increased twitch forces alongside elevated expression of genes governing contractile function. This combination offers the benefit of the viscoelastic feature of shear-thinning, that allows the hydrogel to be readily extruded while preserving its reformed structure after extrusion [100]. Further, multi-extruder bioprinters provide high-throughput modifications, allowing simultaneous printing tasks to boost efficiency and reduce the time in printing complex parts. Nonetheless, reduced graphene oxide has several disadvantages that must be considered in the formulation of bioinks, including inadequate dispersion, limited stability, and the possibility of inducing structural flaws [104]. Sanjuan-Alberte et al. aimed to minimize the mismatch between biological and electronic systems in bioelectronic applications by implementing another strategic combination, which is dECM and multiwalled carbon nanotubes [105]. The nanotubes mimicked both the morphology and conductive features of the dECM fibers and demonstrated that even in the absence of electrical stimulation, the conductive properties of the materials can enhance the contractile behavior of human pluripotent stem-cell derived CMs. PEDOT:PSS was integrated into a collagen-alginate hydrogel and was shown to increase CM length, sarcomeric organization, and Connexin 43 expression which indicated tissue maturation [106].

### 2.4. Heart-on-a-Chip

Conventional methods of 3DCC are static and can result in the accumulation of biochemical waste, especially in the central portion of the constructs, which may adversely affect the cell viability [107]. The development of microfluidic platforms has significantly enhanced our ability to characterize tissues and perform in situ analysis, while also improving the efficiency and precision of drug testing, cardiotoxicity evaluation, and disease modeling. Advantages of these platforms include integration of co-culture environment within the same device, allowing the mimicry of complex cellular environments found in native tissue [108,109]. In addition, microfluidic devices facilitate the generation of exact concentration gradients of pharmaceuticals, enabling researchers to investigate the impact of varied drug concentrations on cells and tissues [10,110]. Through microfluidic systems, several parameters can be monitored and controlled, which include pH level, nutrient supply, oxygen saturation, and temporal drug-dosing [111]. Microfluidic devices can enhance efficiency and throughput by enabling the simultaneous testing of several medications or drug combinations, thereby markedly accelerating the drug screening process relative to conventional approaches [112,113,114,115]. The implementation of automation can enhance the efficiency and speed of these processes. Furthermore, downstream processes significantly reduce immense cost and reagent usage, given the size of the systems.

HoC (Heart-on-a-Chip) models are a subset of microfluidic technology that continues to gain traction. Countless studies have examined their vast potential in addressing the shortcomings associated with traditional preclinical models for recapitulating microscale anatomy and biomechanical performance [116,117,118] (p. 4), however, there are yet to be pre-clinical studies utilizing this technology [119]. Even as of now, the Food and Drug Administration has not approved any type of cellularized 3D-printed tissue for medical purposes [119]. A standout example is the I-Wire platform [120], which is composed of a low-cost mold embedded with wires, and designed to grow 3D cardiac constructs using neonatal rat CMs which can measure mechanical and electrophysiological parameters. Sidorov and colleagues observed that the differentiated neonatal CMs were able to organize themselves and behave similarly to normal heart tissue after molding and 13–15 days of culture. The devised system offers the potential to be low-cost, compact, and up-scaled to medium throughput. To support this, the authors are currently working on compacting the system and implementing a cantilever dedicated to each tissue construct in a well plate.

**Figure 2:**
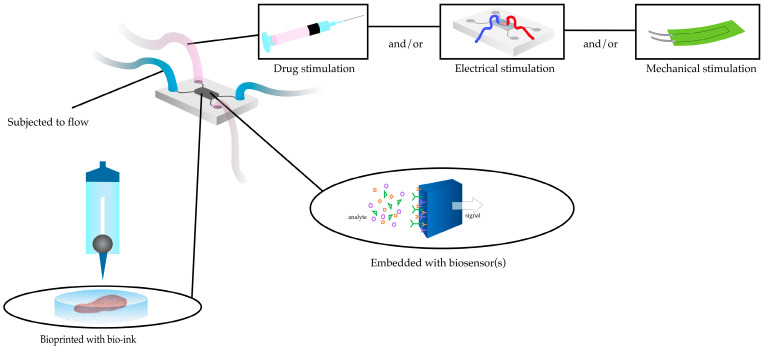
A representation of a possible heart-on-a-chip design embedded with one or more biosensors.

Although research on the integration of microfluidic technology with 3D bioprinting is limited, the subject presents significant opportunities for experimentation given the evident high-throughput benefits of both methodologies. The fabrication of HoC predominantly utilizes 3D printing technology for one or both of two principal functions: the production of the main body of the chip [13] and the generation of the cardiac tissues [83]. Among the studies employing the combined technique of bioprinting and HoC technology, Faulkner-Jones and colleagues [121] built and validated a valve-based cell-printing system, demonstrating that there was no significant impairment of the physiological function of the human induced pluripotent stem cell (hiPSC)-derived CMs compared to the standard manual pipetting cell-culture methods. An emerging approach to streamline the generation and further characterization of the behaviour of EHTs is to combine the advantages of bioprinting cardiac constructs with those of microfluidic devices (Figure 2). For example, subjecting the bioprinted tissue with different flow rates stimulates in vivo conditions for cell maturation. Further stimulation using electrical signals, mechanical force, or even injection of different drugs will alter the state of the tissue to reflect pathological or normal state of the heart. 

In recent work, Zhang et al. introduced a brand-new hybrid approach that combines microfluidic and bioprinting technologies to create endothelialized cardiac tissue [83]. To help endothelial cells migrate toward the tissue’s periphery and contribute to the formation of an endothelium layer, they were able to enclose them in bioprinted microfibrous lattices using microfluidics technology that they previously developed [122]. Another novel method of integrating both microfluidics and 3DBP is done by Lee and colleagues, who reported generating not only a cell-laden scaffold but also gelatin channels suitable for tissue perfusion in a single printing session [71]. Bioprinted HoC systems hold promise in the tissue generation process as they allow replication of complex structures and promote anisotropic alignment, while simultaneously enabling real-time monitoring of contractility, electrophysiology, and cellular responses [121]. For these reasons, HoC models can be employed to generate longer-functioning vascularized heart tissue constructs [83,123] as cellular morphology can be maintained while fluid flow and shear stress are applied. The shear forces can be precisely regulated in a manner to mimic blood flow, enabling the stimulation of nutrient perfusion and cell signaling seen in the human body [124].

Applying suitable mechanical and electrical stimulation has also been shown to support the maturation of cardiac tissues in chip-based models and improve their physiological performance [125,126,127,128]. Capable of integrating various biosensors and allowing for modular design, microfluidic devices provide means to apply mechanical and chemical stimulation independently or together to expedite tissue maturation. Min et al. [110] co-cultured three different types of cardiovascular lineage cells (CMs, ECs, and CFs) derived from hiPSCs and embedded them in a decellularized heart tissue-derived ECM hydrogel within a microfluidic chamber chip. The efficiency from merging the long-term culture on a macroscale (1–1.2 mm) with the application of dynamic medium flow was two-fold, as it created a perfusable culture system that allowed structural and functional maturation of the tissue.

In addition to aiding researchers in comprehending cardiac tissue growth, HoC enables the simulation of flow conditions that occur in vivo during both healthy and pathological states. In 2016, a novel 3D bioprinting-based hybrid approach was introduced [83] to create an endothelialized myocardium for integration into a HoC. A microfibrous scaffold containing ECs was printed and seeded with cardiomyocytes, then embedded into a microfluidic bioreactor adapted from a liver-on-a-chip system for cardiovascular and toxicity testing [129]. The authors reported the translatability of the system to house hiPSC-CMs, although they utilized neonatal rat CMs as a model type for proof-of-concept optimizations. As demonstrated, liver-on-a-chip devices can be adjusted to cardiac applications. The influence of hepatic metabolism on off-target cardiotoxicity within a system comprising both cardiac and hepatic components have been investigated by Oleaga et al. [130]. They constructed a microfluidic device that integrated multielectrode arrays for electrical measurements with silicon cantilevers for mechanical measurements. Illustrating that the liver mitigates the cardiotoxicity of a cardiotoxic parent compound by metabolizing it into non-toxic metabolites, the multi-organ system also simultaneously reduced the cardiotoxicity of a toxic parent compound. The modular design of liver-on-a-chip technology elucidates that the liver functions to either activate a prodrug or metabolize a drug resulting in metabolites that may possess cardioactive or cardiotoxic properties. Despite the promising start of HoCs, further research and throughput enhancement are needed to clarify the detailed pathways involved in drug-related cardiovascular toxicity.

Recent designs delve into incorporating vascularization, increasing facilitation of endothelial network development, sustaining optimal cellular viability and activity, and conducting contractile force analyses using bioprinted HoC models [28,50,53,69]. One of the few vascularization attempts using both bioprinting and microfluidic techniques, by Miller et al. [33], addresses necrosis that occurs in the cores of 3D-engineered tissues, which are densely populated with cells. The authors produced a 3D-printed network lined with ECs and supported by sacrificial carbohydrate filaments, then subjected it to pulsatile and high-pressure flows to mimic a native vascular tree. The authors confirmed that their generated tissues sustained metabolic function and were protected from necrosis.

An alternative method to mitigating necrosis is theoretically feasible by substituting the necrotic area with a cardiac tissue patch. Cardiac patches facilitate the restoration or enhancement of heart muscle activity by offering a framework for new tissue development, transmitting electrical impulses, and administering bioactive substances to encourage healing and regeneration. A patch of this nature could aid in contraction. However, contractile cardiomyocytes exhibit minimal proliferation rates. On the contrary, efforts are underway to develop stem cell solutions that can produce an adequate number of functioning cardiomyocytes for this purpose [131]. In particular, the presence of channels and grooves in bioprinted tissues containing stem cells may enhance cardiac tissue functionality, also referred to as topological or surface guidance. The deliberate topography may beneficially affect the morphology and physiological functions of cells. Tijore et al. bioprinted a cell-aligning hydrogel microchannel scaffold in which the channels and grooves on the surface were observed to enhance the synchronized beating of the heart [132].

### 2.5. Challenges in Integrating 3DBP with HOC

The incorporation of 3D bioprinting onto HoC platforms encounters constraints related to shape, materials, and flow compatibility. The dimensions of several bioprinted constructions (millimetres to centimetres) often surpass the microfluidic channel sizes used in organ-on-a-chip systems, complicating the formation of thick tissues without impeding perfusion or modifying shear circumstances [133]. Secondly, the biomaterials often used in microfluidic chips, such as PDMS, significantly contrast with the soft hydrogel-based bioinks required for cardiac tissues, resulting in discrepancies in stiffness, adhesion, and optical characteristics that hinder integration and real-time sensing [134]. In addition to structural and material problems, functional integration presents additional obstacles. Cardiac tissues necessitate the synchronization of mechanical contraction, electrical conduction, and nutrient/oxygen perfusion; however, integrating sensors and actuators into a printed tissue within a microfluidic chip poses challenges, particularly when the printed hydrogel layer obstructs optical access or interferes with embedded electrodes or sensors [126]. Furthermore, the advancement to higher-throughput formats (many tissue channels per chip, parallel fluidic control) while ensuring consistent cell insertion, alignment, and maturation remains unstandardized, hence limiting the translational potential for drug screening.

## 3. Next-Generation Cardiac Model Systems

### 3.1. Organ Building Blocks

Defined as self-organizing 3D cellular structures, organ-building blocks (OBBs) are designed to replicate in vivo organs on a smaller scale and potentially be used to build or repair entire organs [135]. OBBs that have been developed typically conform to two prevalent structural categories: spheroids and organoids. In contrast, more complex formations, such as assembloids, are constructed through a deliberate amalgamation of smaller OBBs (Figure 2). The overarching goal of OBB systems is to assemble units into larger tissues that can reach up to the size of the organ of interest and simultaneously increase overall complexity. With advantages owing to their intrinsic ability to self-assemble, self-organize, the expedited process of creating larger constructs, potential for prevascularization and cell compaction comparable to native environment [135], OBBs show great promise, especially when coupled with bioprinting technology.

As cells in OBBs are pre-organized into tissue-like microenvironments, bioprinting with OBBs results in faster functional integration and maturation post-printing compared to single-cell suspensions, increasing throughput of macro-scale architectural production [135]. In the past, OBBs have been utilized in sacrificial writing into functional tissue (SWIFT) bioprinting, wherein a sacrificial gelatin ink is extruded into a consolidated slurry (Figure 1F) of cardiomyocyte OBBs, extracellular matrix, and fibroblasts [98]. In the context of cardiac tissue engineering, Skylar-Scott et al. fabricated an OBB matrix comprising spheroids formed with hiPSC-derived cardiomyocytes and cardiac fibroblasts [98], along with extracellular matrix components (Figure 1). The researchers initially developed cardiac constructs featuring one perfusable branch. Following eight days of continuous perfusion, these constructs demonstrated well-defined sarcomeric organization and exhibited approximately a twenty-fold increase in contractile force. Upon validation of this simplified model, the SWIFT bioprinting technique was utilized to create more complex architectures. Utilizing patient-derived anatomical data, the authors constructed a left anterior descending coronary artery network within the cardiac OBB matrix in a 3D printed mold to replicate a myocardial wedge at half-scale.

The interest in OBB systems is emergent yet increasingly becoming the focus of tissue engineering, with an early example being a study that grew toroidal tissue by manually positioning spheroids into rings within a hydrogel [136]. As a proof of concept, this study is among many others that contribute to the intrigue and pursuit of self-assembling bio-ink and the support for using cell aggregates in bioprinting technology. For various advantages such as increased throughput and enhanced tissue mimicry, research on the generation of engineered tissue has recently been investigating the integration of OBBs into bio-ink [137,138,139]. A notable example is when Ahrens et al. [140] created a bioink composed of contractile cardiac OBBs, which were aligned in various patterns and printed into cardiac sheets. Their results indicated that the constructs’ contractile forces and conduction velocities increased and even exceeded those of spheroid-based cardiac tissue.

Furthermore, using patient-specific induced pluripotent stem cells (iPSCs) to make human cardiac organoids or spheroids facilitates the integration of an individual’s genetic information, including all of the loci that could affect how they respond to medication [141,142,143,144,145]. This type of personalized technology enables researchers to observe patient-specific responses to different treatments. Cetnar et al. [146] introduced a first generation of anatomically accurate and functional model of the human heart at various phases of development. Their platform allowed precise modification of microenvironmental factors, like flow and geometry, with ease. The work expanded avenues for exploring developmental processes and their associated disorders.

Utilizing OBBs in biomanufacturing has great potential to address the ongoing obstacles of tissue scalability, since conventional EHT generation methods are laborious, lengthy, costly, and are prone to having batch variation [147,148]. Specifically, the generation of autologous human organs requires 10–100 billion patient-specific cells [148], which is challenging to manually expand in culture unless done in a bioreactor. To date, addressing the challenges in OBB production and assessments (i.e., throughput) is being done in multiple ways, such as combining current generation techniques [149,150], integrating microfluidics, and bioprinting technology [123,151,152,153,154]

### 3.2. Spheroids

**Figure 3:**
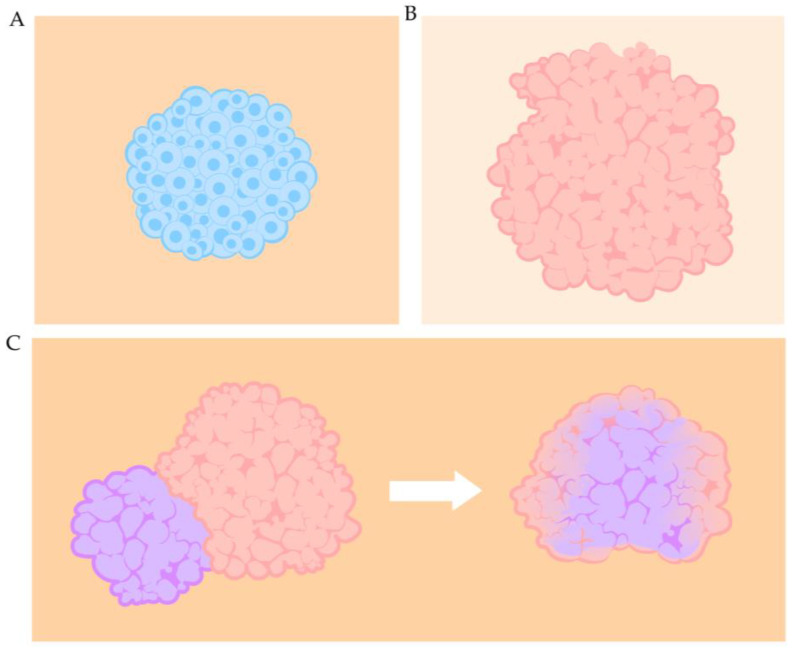
A diagram of the main types of OBBs. (**A**) A cardiac spheroid, (**B**) a cardiac organoid, and (**C**) the fusion of two types of organoids (cardiac and brain) into one type of assembloid.

In general, spheroid cell culture is a comparatively simpler and quicker model system for drug screening and basic cellular behaviour studies than organoid culture, with advantages lying in the ease of generation and lower cost as opposed to organoid models. Cardiac spheroids are advantageous for pharmacological testing and are simple to produce; nonetheless, they lack the directional properties inherent to healthy myocardium, which are crucial for maintaining the functionality of modified cardiac tissues over time. Further, due to the inherent self-adhesion properties of cells leading to spheroid compaction, the structure of spheroids more closely resembles solid tumors (Figure 1M and Figure 3B), with outer proliferative layers, intermediate quiescent layers, and inner hypoxic cores [84,90,91].

Bioprinting with spheroids can be employed via multiple techniques. Zhao et al. [87] utilized a microfluidic nozzle filled with cells to generate a spiral-shaped droplet by airflow-induced rotation. The authors found that gravitational pull contributes to the distribution of spheroids into a CaCl_2_ (2% *w*/*v*) solution as a final cross-linking step when utilizing sodium alginate-based bioink. A more direct approach utilized hybrid printing technologies such as CELLINK BioX and the Advanced BioAssemblyBot^®^400 (BAB400) system to produce spheroidal droplets made with human CMs and CFs [92]. The research indicated the presence of both open and closed pores, which facilitated nutrient transfer and improved mechanical integrity. The Kenzan method is a sophisticated and innovative approach in Japanese bioprinting technologies wherein needle arrays are employed for spheroid placement [135]. However, the method is constrained by various limitations such as low throughput (as it assembles spheroids individually at a time), the fixed needle configuration which restricts its versatility, and the impact of maturation speed and tissue fusion from removal of the needle [40]. Furthermore, fused tissue spheroids exhibit significant contraction and compaction, leading to dense packing and the development of a pseudo-capsule. This phenomenon diminishes the diffusion and accessibility of oxygen and nutrients, along with the export of metabolic waste products.

A study in 2017 [93] detailed the promise of using cardiac spheroids as a model to study the human heart microenvironment, by co-culturing iPSC-derived CMs with ECs and CFs. The results showed that culture modifications, preventing hypoxia while simultaneously promoting the growth and maturation, allowed the cellular organization to resemble that of the human heart. Further validation on whether the multi-type cardiac cell spheroids could be used as models to treat human heart toxicology, was done using increasing concentrations of doxorubicin, and proved the role of CFs in doxorubicin-induced apoptosis of cardiomyocytes. So far, there is an increasing utilization of cardiac spheroids for biofabrication and regenerative medicine as new technologies advance to improve their generation and applications [38,90,94,95,96].

### 3.3. Organoids

Cardiac organoids (cardioids) are self-assembled 3D model systems grown from progenitor or stem cells (Figure 3B), designed to replicate key structural and functional features of the native heart system. The first cardioids were attempted by Hofbauer et al. in 2021 [17], in which the tissues underwent self-directed specification and spatial organization to form chamber-like architectures with internal cavities. In a later study, Mendjan and colleagues expanded the platform to replicate the development of all major embryonic heart compartments, which included the ventricles, atria, outflow tract, and atrioventricular canal [155]. This platform was able to generate region-specific gene expression patterns, structures, and functions that closely resemble in vivo physiology. As of now, only a handful of studies have been conducted to develop and/or use cardioids [16,156,157]. For instance, Wang et al. and Lewis-Israeli et al. generated cardiac cells that developed into an organized muscle architecture and possessed functional traits typical of native fetal cardiac tissue [16,158]. However, the development of cardioids remains inchoate compared to other organoid systems, which may be attributed to the intricate relationship between the heart microenvironment and its architecture. While cardioid research has more room to grow and improve, researchers have recognized that organoid technology opens up more effective therapeutic strategies, such as downstream testing in drug development to enhance the precision and efficiency of drugs [159].

### 3.4. Assembloids

Multi-organoid constructs, also termed assembloids, consist of more than one type of organoid (Figure 3C). The significance of synthesizing multiple types of organoids together stems from studies noting that organs operate with extensive cross-talk among each other [160,161,162]. Utilizing these multi-organ models will be key for investigating the interactions between the heart and the other organs. In constructing cardiac assembloids, the complexity of the construct is increased significantly compared to single organoid systems, as new properties emerge, including changes in cell-fate specification, morphology, and enhanced maturation [163]. O’hern et al. presented a model of iPSC-derived human heart assembloids to demonstrate the successful integration of neural crest cells (NCC) into the human embryonic heart in vitro [164]. Since NCCs are involved in the development of the human heart, the authors surveyed their activity during relevant developmental stages and confirmed that the NCCs took part in the assembloid maturation process. In particular, their study showed that the NCCs migrated and differentiated into neurons that could generate impulses and mature into glial cells. Daley et al. created a bioprinted assembloid model consisting of fused spheroids within a self-healing hydrogel that promoted high cell-density heterogeneity [165]. Basing the system on scarred cardiac tissue which resulted from myocardial infarction, the authors were able to replicate both structural and behavioral features and examine the effect of pro-generative microRNA treatment on tissue healing. Other assembloid systems, such as cardio-cerebral, cardio-pulmonary, cardio-renal, and cardio-intestinal systems, have been developed as well [166,167,168,169,170,171,172].

### 3.5. Challenges and Strategies of Organ Building Block Systems

In organ-building block systems, the lack of functional vasculature to supply nutrients and other trophic factors limits the constructs from reaching their full potential [163]. Pre-vascularization techniques were attempted and have shown an increase in the survival and implantation of cardiac cells under ischemia [173]. As such, when these steps are combined to increase the complexity of the constructs, they are better positioned to replicate the structure and function of the heart. The generation of the multi-component tissue culture method offers a more streamlined approach, leading researchers closer to rigorous testing of the tissues.

OBB systems still face a number of technical and analytical challenges despite these impressive advancements. A common challenge with traditional techniques for analyzing OBBs is enhancing the capacity to accurately evaluate their mechanical characteristics in real time and in situ [159]. A significant obstacle is the need to meticulously and dependably evaluate the structure and function of these intricate builds. As OBB-based tissues are able to achieve increased cellular variety and architectural complexity, conventional evaluation methods often lack the depth and resolution for accurate characterization. Consequently, there is a call for innovation in tissue imaging equipment and analytical tools. The demand for non-destructive, real-time analysis has catalyzed the creation of sophisticated imaging technologies that can temporally monitor live structures. Moreover, the production of tools with refined precision, a greater understanding of the mechanobiology, as well as standardized protocols are required in order the overcome the challenges. In the last few years, novel advancements, such as the incorporation of biosensors in OBB technology, have been introduced and are rapidly evolving in the field [13,174,175,176,177,178,179,180]. A rising method to monitor and analyze an OBB system with promising capability to increase throughput is by using an artificial intelligence-driven process created using machine learning, manifold learning, unsupervised data clustering, and ensemble learning. Kowalczewski et al. [181] explained that their system can monitor calcium uptake, which is a key process that characterizes the identity and function of an organoid. This new technology can be used to decrease batch variability and facilitate the integration of further design when generating organoids [181]. Utilizing OBBs as modular components for 3DBP has significant potential for scalable organ-specific tissue engineering. Significant obstacles hinder their translation: producing highly uniform organoid bioprinted bodies (OBBs) at scale is challenging, and upon assembly into bulk constructs, efficient vascularization presents a bottleneck—embedded vascular networks are necessary for tissue volumes exceeding 1 mm^3^ but are difficult to implement [148]. Moreover, the incorporation of OBBs into printed architectures encounters structural and functional challenges: preserving accurate spatial alignment without deformation, ensuring the integration of OBBs into a unified tissue (preventing inter-block conduction and force barriers), and scaling processes for high throughput while adhering to quality control standards remain unresolved [135].

Other ways of evaluating cardiac OBB systems employ traditional imaging practices, which include fluorescent markers and light penetration. However, these methods are limited by the depth of tissue penetration, photobleaching, and autofluorescence, which additively decrease the image quality. On the other hand, tissue-slicing techniques are advantageous in maintaining tissue structure and enabling high-resolution microscopic analysis, but are a highly time-consuming and arduous process that is prone to tissue damage, and thus unusable data [159]. A small number of imaging systems were developed to address these ongoing obstacles [182,183]. For instance, Optical Coherence Tomography (OCT) can be combined with fluorescence microscopy in a system that is non-invasive, high in spatial-temporal resolution, and such a system can monitor the internal 3D structure of beating cardioids in real-time [184]. Other custom OCT systems were developed, wherein non-invasive monitoring can be done without fluorescent labeling to perform longitudinal morphological and functional characterization of human cardioids [185]. Ming et al. were the first group to report the formation of structurally diverse cardiac chambers in organoids, with the emergence of complex connections on day four of differentiation [185].

Moreover, an attempt to increase the efficiency of the generation and analysis processes was made by Seguret et al., who created an assay that enables the rapid generation of 3D cardiac loop tissues within a 96-well plate format [186]. The assay utilizes a transparent, flexible polymer-based central post, allowing real-time assessment of contractile force and tissue movement, thereby improving experimental consistency and standardization. We predict that recent developments in extracellular recordings, as well as flexible electrodes, will likely allow for long-term, non-invasive and scalable electrophysiological studies of assembloids [163].

## 4. High-Throughput Screening of the Bioprinted Cardiac Tissue Models

**Figure 4:**
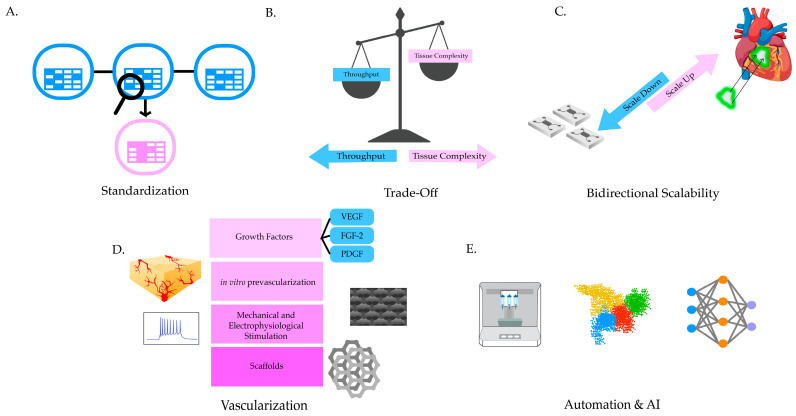
Considerations for improving high throughput processes in cardiac engineering, partially created using BioRender (https://www.biorender.com/). (**A**) Standardization through the verification of outcomes across several data sets or investigations, followed by the synthesis of the validated information. (**B**) Scaling down tissues to microsize allows more tests to be performed simultaneously at lower cost, while scaling up tissues improves smooth integration of cardiac implants. (**C**) Different vascularization strategies can be employed such as adding growth factors, prevascularizing the tissue prior to implantation or fusion with host tissue, mechanical and electrophysiological stimulation, or by planting angiogenic cells on biomaterial-derived scaffolds. (**D**) Automation of tissue generation can be done using a bioprinter, and analysis via machine learning processes of a designed algorithm for faster diagnoses, and discovery of new insights of cardiac disease and function. (**E**) Operational throughput and biological complexity exist at opposite ends of the design spectrum since prioritizing one compromises the other.

One of the main goals for effective and efficient production and evaluation of cardiac tissue models is to expand the scalability range while maintaining, or even increasing, the tissue complexity and maturation for both research and clinical applications. Numerous studies have bioprinted cardiac tissue models utilizing combinations of existing methods in novel ways, but there is a lack of standardized sets of parameters to benchmark against [183,187,188,189,190]. In most studies, these parameters rely on rough estimations of in vivo heart properties, which include, but are not limited to, myocardium thickness, flow velocities, and cell-type population ratios [191,192]. As simple as it sounds, developing standardization is a formidable opponent, especially when genetic and demographic diversity among cell lines leads to variations in baseline contractility and introduces batch-related inconsistencies [193,194,195]. For standardization to be established within cardiac tissue engineering, moderation between throughput and biological intricacy must be optimized to a sufficient level, such that the generated constructs can be readily tested with consistent results. To ensure the repeatability and comparability of the engineered tissue systems, a regimented process must be established, verified, and implemented at each stage, including material supply and process, data collection, validation, and reporting (Figure 4A). At this point, an inverse relationship exists between operational throughput and biological system complexity [89,196,197] (Figure 4B), as an increase in production may risk the loss of key characteristics of cardiac tissue, and a heavy focus on tissue maturation challenges the production of multiple constructs.

Although scaling down tissue sizes is logical for high-throughput applications, compatibility issues are likely to arise in downstream assays, such as histology, since adjustments to protocols or equipment need to be made and optimized to accommodate the samples and reduce sample loss. On the other hand, sizing up EHTs is met with a range of other problems, such as those associated with the limited proliferative capacity of CMs and poor vascularization [89]. Hence, bilateral scalability (Figure 4C) is a valuable tool and merits consideration for increasing throughput in clinical and industrial applications.

The degree of structural and functional development in current cardiac tissue models still falls short compared to that of the human heart, especially in areas such as electrophysiology, biomechanics, and metabolism. These limitations collectively decrease the fidelity in pathophysiology within the constructs for both healthy and diseased cardiac tissue models [159].

Vascularization strategies (Figure 4D) have been attempted not only to address necrotic behaviour in scale-up procedures, but also to facilitate further structural development, biochemical, and electrophysiological function [173,198]. Vascularization enhances throughput by more effectively supplying nutrients and expelling waste via the tissue, hence facilitating maturation. Pre-vascularization of the tissue constructs is also emerging as an effective method for the long-term objective of seamlessly integrating them with native tissue for implantation [199,200,201].

Furthermore, utilizing automation, robots, and machine learning opens up avenues to streamlining processes such as data collection and analysis (Figure 4E) [202,203,204]. Notably, real-time quantification of processes and for predictive toxicology modelling has been ventured into by Zhu and colleagues [205]. The group tackled reproducibility issues by systemically examining and analyzing 32,000 spheroid photos to determine the influential elements that impact the reliability of the 3D model. Their findings suggest ways to standardize 3D culture methods for consistency, which would increase their utility in drug testing, customized medicine, and tumor biology. Other ways of streamlining processes include the introduction of modular bioreactor systems [206,207,208], which allows researchers to finely tune environmental variables for parallel culture conditions, thereby reducing batch-to-batch variability and enhancing reproducibility. Simultaneous work on adaptive bioinformatics pipeline time will facilitate researchers to parse and obtain key information from substantial datasets that would provide detailed analyses of tissue maturation, function and drug response. Overall, the coalescence of novel innovations or approaches that combine existing techniques with rigorous quality-control mechanisms is key to reaching the point where 3D engineered tissue can fully replace 2D models for disease modeling [89].

## 5. Transcriptomics for High-Throughput Improvement in Analysis of 3D Cardiac Tissue

In generating 3D cardiac tissue constructs, understanding the processes for cardiac development and regeneration is critical, and thus the importance of transcriptomic methods is underscored. Cardiac morphogenesis can be further understood using single-cell RNA sequencing (scRNA-seq) and single-nuclei RNA sequencing (snRNA), which allow global transcriptome profiling at the single-cell resolution. From the sequencing data, developmental processes, electrophysiological phenotypes, immunology and pathology can be scoped and even simulated computationally to predict cell behaviour after the treatment of drugs [209,210,211]. However, few transcriptome-wide scRNAseq studies investigate the spatial and/or temporal distributions of cardiac gene expression. Hence, there is a need for innovative strategies to provide essential details regarding the locations of several cell types involved in the maturation of cardiac tissue [212]. Within the realm of RNA sequencing, spatial transcriptomics (ST) addresses the lack of spatial contextual information of the cells, such as the interactions and relationships among other cell types. By quantifying the number of gene transcripts at designated locations within a tissue sample, ST is able to utilize this data to create spatial maps of gene expression to reveal cell organization. When both spatial and temporal gene differentiation data are combined, researchers are able to delve into the positional context of the underlying architecture, metabolic activity, and cell fate within developing tissue. Notably, Asp et al. [213] devised a molecular method that combined three different sequencing technologies (Next-Generation Sequencing-based ST, scRNA-seq, and in situ-based ST) to map out different cell types occupying the embryonic heart at three developmental stages, which were 4.5–5, 6.5, and 9 weeks post-conception. Their results were validated by the identification of human embryonic cardiac cell types by sc-RNA seq, while the temporal data was explored using the Next-Generation Sequencing-based ST. The group reported the early establishment and the maintenance of spatial gene expression across all of the stages of development. Moreover, expression patterns were able to be identified across different cell types, including those recently identified as contributing to cardiac development.

According to Nguyen et al. and Farah et al. [214,215] two main methods can be identified from ST technology: sequencing-based methods [216] that focus on mapping out spatial locations using genetic barcodes, and imaging-based technologies [148] that identify RNA in situ via microscopy. Fluorescence In Situ Hybridization (FISH) is a type of imaging-based ST technique that uses fluorescent probes to bind complementary RNA sequences, allowing each transcript to be visualized. Litviňuková et al. used multiplex single-molecule fluorescence in situ hybridization (smFISH), sc-RNA seq, and snRNA seq [217] to identify the molecular markers, intercellular networks, morphological occurrences, and the relationships between different cell types and their environment. In particular, the study explored skeletal muscle and kidney transcriptomes and compared them with cardiac-specific cell signatures, thereby improving current understanding of the human heart and serving as a reference for future investigations involving multicellular interactions involving cardiac cells. An example using an imaging-based method, presented by Lázár et al. on bioRxiv [218], presents a comprehensive spatial map of different cell types and their gene expression patterns during early cardiogenesis, furthering the understanding of the mechanisms underlying heart development and the potential genetic links to heart disease. Additionally, the authors detail the development of cardiac autonomic innervation and provide the first spatial representation of chromaffin cells in the fetal human heart. In contrast to imaging techniques that necessitate multiple cycles of staining, imaging, and stripping, ST methods enable the acquisition of multiplex data in a single run, significantly enhancing throughput.

ST techniques vary in resolution, with throughput being contingent upon the total capture area [215]. Recent techniques have demonstrated higher resolution and throughput of capture procedures. For example, Slide-seq is a sequencing method that detects beads, each uniquely barcoded, on the surface of a tissue slice [219]. The beads capture the RNA from different locations of the tissue, with the specific origin of each sequence determined by the distinct barcodes. An enhanced version of Slide-seq is High-Density Spatial Transcriptomics (HDST), which yields higher resolution and more detailed analysis of genes at a subcellular scale [220]. Neither Slide-seq nor HDST has been employed to assess cardiac tissue; however, their high resolution for analytics suggests potential applicability in this area. Moreover, there is a rise in the number of studies using other ST techniques in human cardiac tissues, dating from 2022 to 2024 [213,215,218,221,222,223,224,225,226,227], indicating that ST is a trending method to reliably view gene expression across multiple sections of 3D tissue. Ultimately, ST bridges the gap between molecular data and tissue structure, enabling a more faithful analysis of 3D cardiac tissue organization and dynamics.

## 6. Conclusions

New research frontiers are increasingly using integrative methodologies that combine existing tissue generation techniques and/or analysis processes which include but are not limited to high-precision imaging, electrophysiological assessments, and functional tests to provide a thorough analysis of tissue health and functionality. The interplay of these technologies will bring revolutionary changes to fundamental and translational research in tissue engineering, offering innovative pathways to understand development and disease, assess pharmaceuticals, and improve regenerative methodologies. As engineered tissues increasingly continue to improve on replicating the multicellular environment and dynamic functions of the human heart, the criteria, equipment and methods of evaluating their maturation, functionality, and integration must also advance. Striking a balance between operational throughput and biological complexity is also essential for progress within the field of cardiac tissue engineering. Accordingly, the advancements towards this equilibrium will undoubtedly require integrating rigorous testing within each step of an engineered solution. As the sector keeps changing, integrating standardization within the processes by sharing benchmark data and establishing consensus around best practices will be critical to increasing overall throughput. Adopting these shared standards would help close the gaps in scalability and complexity that currently exist and transitioning cardiac tissue engineering from being an experimental novelty to having clinical utility.

## Figures and Tables

**Table 1:** Comparison between the three main types of bioprinting techniques for tissue engineering.

Technique	Resolution	Range of Compatible Material	Scalability	Advantages	Limitations
Extrusion-Based Bioprinting	200–500 µm filament precision [32]	Broad (viscous, cell-dense bioinks) [33]	Moderate (can be adjusted with automation)	Suitable for facilitating anisotropic filament alignment [34], supports high cell density and scalability [35], automation capability [33], ease of operation [35], affordable [33]	Shear stress can cause damage to cells, difficult to get lower resolution, needle clogging [35,36]
Laser-Assisted Bioprinting	20–100 µm [37,38]	Low viscosity bioinks [39]	Low; suitable for patterning but limited in bulk tissue [40]	Relatively fast deposition, high cell viability, heterocellular patterning, multi-material printing [38], nozzle-free technique that prevents clumps [41]	Weak in mechanical stability, can cause thermal damage to cells [39]
Stereolithography/Digital Light Processing	10 [42]–50 µm [43] (DLP)	Photo-cross linkable bioinks	High; may slow down with larger constructs	High speed and fidelity, suitable for complex microfeatures [43], large volume of initial bioink required [44]	Curing by UV can be detrimental to cells, light penetration is limited to the thickness of the construct, larger and softer structures are prone to deformation [43]

## Abbreviations

The following abbreviations are used in this manuscript:

3DCC	3-Dimensional Cell Culture
3DBP	3-Dimensional Bioprinting
BAB400	BioAssemblyBot^®^400
HTS	High-Throughput Screening
CVD	Cardiovascular Diseases
dECM	Decellularized Extracellular Matrix
CAD	Computer-aided Design
CEVIC	Continuously Extruded Variable Internal Channeling
DLP	Digital Light Processing
EBB	Extrusion-Based Bioprinting
EHT	Engineered Heart Tissue
FRESH	Freeform Reversible Embedding of Suspended Hydrogels
HoC	Heart-on-a-Chip
CM	Cardiomyocytes
iPSC	Induced Pluripotent Stem Cell
hiPSC	Human Induced Pluripotent Stem Cell
CF	Cardiofibroblast
EC	Endothelial Cell
ECM	Extracellular Matrix
LBB	Laser-Assisted Bioprinting
OBB	Organ Building Block
NCC	Neural Crest Cells
OCT	Optical Coherence Tomography
PEDOT:PSS	Poly(3,4-ethylenedioxythiophene) polystyrene sulfonate
PEG	Poly(ethylene) Glycol
rGO	Reduced Graphene Oxide
sc-RNA Seq	Single-Cell Ribonucleic Acid Sequencing
SLA	Stereolithography
Sn-RNA seq	Single-Nuclei Ribonucleic Acid Sequencing
ST	Spatial transcriptomics
FISH	Fluorescence In Situ Hybridization
smFISH	Multiplex Single-Molecule Fluorescence In Situ Hybridization
HDST	High-Density Spatial Transcriptomics
UV	Ultraviolet
